# 1892

**Published:** 1893-01-07

**Authors:** 


### 1892.

THE progress of the medical sciences becomes more and more interesting every year, and the scope of those sciences tends more and more to include all that is proper to man. The latest illustration of this identification of medicine with man is furnished by the recent work of Professor Kraft Ebing, of the Vienna University. Professor Ebing, as some will think, has discovered a new disease. The more philosophical will perceive, however, that he has not so much discovered a new disease as given a new name to an old one. "Political mania" is the addition which the Vienna Professor has made to medical terminology.

### POLITICAL MANIA.

"History," says Professor Kraft Ebing, "especially contemporary history, tells of numerous individuals who, dissatisfied with the conditions of human society, believe themselves called upon to better mankind; or, at least, to contribute towards the improvement of the race by putting something new in place of the old. Such persons generally either act under the suggestive influence of others, or lose their mental equilibrium in consequence of the continual arrival of exciting news, generally in eventful rather than in quiet times. Being thus hurried on to act, they appear in the *rôle* of public tribunes, leaders of plots and insurrections, founders of sects and new political parties.

"The masses, though not equally unhinged, are drawn away by the same outward influences, to follow them like sheep, and to remain under the spell of their eloquence, which is nearly always original or eccentric, and sometimes fanatical. These men are usually mentally afflicted, and their ultimate fate is maniacal exaltation; but, long before that is recognised, they generally succeed in instigating their fellow citizens against each other, in setting class against class, race against race, and the climax of their fanaticism takes the form of dynamite attempts and political murders, simply because they lack the reason to understand that the removal of the representative of a certain system does not destroy the system itself, that rebellion of any sort calls forth reaction, and that social progress demands a gradual evolution."

There is much that is true in this, and much that is false. Physical science is apt to think that it alone is worthy of the name of science, because the phenomena with which it deals are gross and objective, and appeal to the senses. This is a view of science worthy only of undisciplined children. Political science, mental science, moral science, are all as well worthy of the name of science as physical science. All that it is necessary to insist upon is that methods which are truly scientific shall be alone employed in the collection and criticism of facts. The names of Moses, Mohammed, Adam Smith, Mill, and Herbert Spencer were known before Professor Ebing burst his shell, and will probably continue to be known long after his name is forgotten. Medicine strives to take the whole man within its purview, and therein she is wise. But she only glances at the whole man, in order that, like the biologist who takes in the whole of nature, she may the more justly comprehend her own special department. Political maniacs there may be and doubtless are, but those persons who are political maniacs are not maniacs because of their politics, but because of the condition of their brains; and they would be just as much maniacs if they were lawyers or physicians as they are when they are politicians. "Common sense" is a saving grace for specialists as well as for other people. Has Professor Kraft Ebing ever considered the question of "war-mania," or measured the sanity of those who make war?

### THE LATE DUKE OF CLARENCE.

The year opened with a public calamity that fell within the province of medicine, but which, unhappily, was of a nature that our science has not yet succeeded in averting with certainty. The late Duke of Clarence was stricken down by a severe attack of pneumonia, and in spite of everything which the most competent medical science and experience of the times could do, he died at the very opening of his manhood's life. It is not too much to say that the whole profession of medicine felt this calamity like a painful blow. The nation sympathised with the Royal Family; the medical profession sympathised as truly with the Royal Family; but it included Dr. Broadbent and his colleague within sympathies almost as deep. Time, the healer, has brought balm to even those sore wounds.

### A PERSECUTED HOSPITAL.

Early in the year the London Hospital seemed to be almost entirely given over to its enemies. Unfortunately a leading daily paper, failing altogether to take a just and comprehensive view of the facts of the case, made itself the mouthpiece of faction, ignorance, and spite. We refer to the subject now for one purpose, and for one only. It is to point out the serious nature of the mischief which resulted to the poor of East London. At a meeting of the Court of Governors held in March, Mr. Murray Ind announced that there had been a falling off in the income of the hospital for the preceding year of no less a sum than £10,000. But let readers consider who those people were who were deprived of the use of this £10,000; they were not the hospital officials, but the sick and helpless poor of the East-end of London. The great hospital of Whitechapel, after exhaustive investigation, was entirely cleared of all blame. But when we weigh the fact of the discontent of one or two inexperienced girl-nurses against the deprivation and suffering unnecessarily inflicted upon several hundreds of sick and helpless men, women, and children by the loss of this £10,000, we may well hold up our hands in pure astonishment. Are we too bold when we ask that on such great public questions as the character of leading London hospitals newspaper editors and other persons of influence will use a careful and conscientious discrimination before committing themselves to courses which lead to such disastrous results?

### "CURES."

The year has seen a development of medical practice which men of unsophisticated minds cannot regard with satisfaction, and which the public ought to have sense enough to disapprove. "Secret" or "specific" "cures" have been adopted or "discovered" by legally qualified medical men. The "gold cure for drunkenness" and the "Mattei cure for cancer" are of this nature. "Hypnotism" may also be included. It is not making any great claim upon the public faith and forbearance when we affirm that the medical profession is the proper tribunal before which all "cures" should be brought, at least in the first instance. We do not consider it so much as possible that the medical profession of the present day would or could deliberately refuse to consider and to adopt any cure, of any kind soever, from any source whatever, which had proved itself to be a real and actual "cure" for any human ill. If it were possible, and should really take place in fact, that medicine as a profession should so utterly fail to understand its duty to the public, then by all means let appeal be made to the natural humane instinct of the whole people. But the very opposite is the true fact. Medical men sacrifice pleasure, health, and in some cases even life itself, in the persistent attempt to extend the bounds of knowledge and to ameliorate the ills of man. Are these the men to deliberately and wickedly shut their eyes to any new truth which may forward the one purpose of their lives? The proper attitude of the public towards the medical profession on this point ought to be one of profound gratitude, because that profession acts as a detector of shams and lies, and so protects the ignorant and credulous from being swindled in pocket and ruined in health by unscrupulous knaves.

### Phagocytosis and Inoculation.

In the region of open and honourable medicine there have been no fresh discoveries of a startling or sphere-resounding nature. No new Koch has risen upon the medical horizon. But scientific progress of the most wide-reaching significance has, nevertheless, continued to be made. The science of microbiology is steadily advancing. The causes and nature of infectious diseases are day by day becoming more clearly perceived and comprehended: and infectious diseases include the greater part of all diseases. The forces of resistance are also being more justly weighed. The doctrine of "phagocytosis" still continues to hold its ground, and to strengthen its credit with those who are most competent to estimate its value. In this connection the names of Lister, Metchnikoff, Hankin, and many others are worthy of special record. The inevitable corollary of a microbic pathology is inocu ation. This, at any rate, seems to be the case according to our present interpretation of facts. Given an infinitesimal microbe, which may at any moment be introduced into the human body by unseen and undreamt-of ways, and which, when introduced, has the power of setting up dangerous or fatal disease, it is obvious that the first of all things to be done is to fortify the human body against the attacks of such a secret and deadly foe. Inoculation is the one method which appeals to the scientific mind, This method may, in time, be so perfected that its use will become spontaneous among educated people all the world over; or it may, sooner or later, be altogether discredited, and give place to simpler, truer, and less repulsive prophylactics. In the present state of our knowledge, he who would prophesy definitely on this subject would be a very foolish person indeed.

### Cholera.

It would be difficult to find any set of facts which more clearly show the miserably slow progress of science, even in modern times, than the history of the cholera epidemic at Hamburg and other Continental towns during the past summer. Physiology teaches by object lessons. But even object lessons require a certain amount of mental capacity and culture on the part of the learner. Nothing has been more convincingly demonstrated than the facts that pure air, cleanliness, and wholesome feeding are the great conservators of life. But Hamburg, the city of commerce, of learning, and of civic freedom for ages and generations, was found by the invading cholera to be a city of ignorance, unscientific stupidity, and every conceivable kind of physical and moral filth. Hamburg has paid the price of mental incompetence. She will, in all probability, pay a still higher price before the current year comes to an end. This is the object lesson which has been presented to English as well as to Continental eyes. We have all seen it! How many of us have in any way laid it to heart? The only persons who seem to have profited by this object lesson are the medical men of our country. They have already stripped themselves for fight. This, too, is an object lesson which the people of Great Britain should ponder. The medical officers of health of our seaports and inland places met in conference at the Mansion House the other day on this very subject of cholera. Did they meet for their pleasure or because their minds were aroused and alarmed! They met because they saw, as from a mountain summit, a deadly enemy in the near distance. They met in order that they might prepare to meet the foe. What they did every Englishman in his measure ought to do in his own home. He, too, without an hour's delay, should prepare his house and his family for a dangerous invasion of cholera.

### "Suffering London."

There has been a reaction in the charitable world during the past year in favour of known sufferers, and methods of benevolence which have the merit of being "old" and "tried." Persistent efforts have been made by the conductors of this journal and by other agencies to bring about that reaction. A year ago we asked the public to consider "London without Hospitals." A few months later, "Suffering London," written by Mr. Egmont Hake, and with an introduction by Mr. Walter Besant, was published. It seems more than probable that these and other efforts have wrought a change in the public mind. For some months there has been an unanimity of kindness among newspaper writers towards hospitals and similar charities. It is possible that as yet no great inflow of funds has been observed. But it is to be remembered that profits in almost every department of business have been unusually meagre. The agricultural interest is driven almost to despair by "bad times," and general commerce has little to offer but complaints. There is no doubt, however, that when the ebbing tide of prosperity begins to flow again, hospitals will be the first of all institutions to welcome the charitable argosies of genial and smiling wealth.

### The Royal Commission.

The Select Committee appointed to deal with the methods and resources of the Metropolitan Hospitals, concluded its sittings, and issued its report during the past year. It must be said of that Committee that it was "as fair as fair could be." It listened to everybody, both great and small, both pertinent and inconsequent. Its patience was inexhaustible. It weighed the evidence, too, with infinite judgment and care. There was but one conclusion to which the committee could come, and to that inevitable conclusion it came in due time. The hospitals, it decided, were human institutions, conducted by human creatures, and therefore liable to mistakes and errors of judgment; but that, as a matter of fact, they were well and economically conducted, admirable in their work and usefulness, and worthy in every respect of the confidence and support of the public. To this we have nothing to add, except that on such an unexceptionable certificate of merit the public should hasten to endorse the conclusion of the Committee by a generous outpouring of its kindly charity.

### A Generous Nurse.

There is nothing particularly new to report about the business of the Royal National Pension Fund for Nurses. It pursues the even tenor of its way, and is steadily successful. One incident has happened during the year which gives pleasure to all who hear of it. Miss Emma Durham, Matron of Scio House, Isle of Wight, has made over to the Junius S. Morgan Benevolent Fund the sum of £200. Nurse Durham had charge of the late Lord Tennyson for a period of nearly two years, and the whole of her earnings during that time she has devoted to the sole use of poor nurses in connection with this benevolent department of the Pension Fund.

### M. Pasteur's Seventieth Birthday.

All men have agreed to honour M. Pasteur; all at any rate but a very few. Even those who do not love Pasteur's methods of work might at least accord him the regard due to high mental capacity devoted to noble ends. In the words addressed to M. Pasteur by M. Dupuy, Minister of Public Instruction—" You had the faculty of being able to concentrate your thought on one subject, and of holding it there for days, for months, for years, a supreme faculty reflected in your face, a creative power of which posterity will read the expression on this medallion, where the artist has traced with your features something of your soul. With the same clearness we read that profound faith in science, that apostle's faith which has sustained you in your career to bear up against all the pains of doubt and discouragement. It should be said to all the world that while you are endowed with the critical sense indispensable for the *savant*, you have in you nothing of the sceptic. Throughout you have had conviction, I may even say faith, mother of high thoughts and immortal works."

### De Omnibus Rebus.

The life and activity of a single year of modern medicine offer an almost infinite variety of subjects for the consideration of the critical and appreciative mind. Space, however, is limited, as may also be the reader's patience. Professor Lombroso discussed "Genius and Insanity" during the year. Brilliant he may have been: sober and sensible he certainly was not. Mr. A. J. Balfour discoursed upon a "Close Time" for politicians, with much physiological wisdom and political geniality. Viscount Middleton tried to persuade the House of Lords to appoint a commission to consider the question of the London fogs. The befogged peers declined to enter upon the dark and dreary topic. One or two new cancer cures were announced from the Continent; but they were not able to get beyond the preliminary "flourish of trumpets" stage. "Anæsthetics" have been laboured a good deal during the year by various persons, but unfortunately very little ground has been gained. It is also to be regretted that no practical steps have yet been taken towards the better education of midwives. Hypnotism and hypnotisers have steadily lost ground. Vaccination has as steadily gained in the estimation of those most competent to judge. Vivisection has been illuminated from various quarters; and the storms which have been raised have very decidedly cleared the air. Professor Hankin has had himself inoculated with the cholera virus; and M. Haffkine, with other youthful enthusiasts, have swallowed cholera fluids in their native habitat. No great harm has come to any of these adventurous persons. Certain physiological experiments have been made by enthusiastic physiologists at Oxford on their own bodies. The experimenters still survive. Dr. Broadbent pointed out to the students at Manchester the new region upon which the science of pathology must now enter. Sir James Paget gently, but very decidedly, hinted to scientific "researchers" that if they would have a little less contempt for "practice" they themselves would be none the worse, and the world would be all the better. Mr. Erichsen made a pathetic appeal for the disestablishment of the sectarian conglomeration of teaching institutions which goes by the name of the "London Medical School," and the founding of a real "London School of Medicine," which shall be worthy of the science of the times and of the greatest city in the world. The British Institute of Preventive Medicine has made another appeal for funds, which ought to command the generous consideration of every intelligent person. The medical world, indeed, is full of scientific and human vitality, and is devoting itself unceasingly to the most ardent pursuit of knowledge for the greatest and noblest ends.

